# Impact of gastrointestinal events on patient-reported outcomes in Asia-Pacific women with osteoporosis: baseline results of the MUSIC OS-AP study

**DOI:** 10.1007/s11657-017-0350-3

**Published:** 2017-07-17

**Authors:** A. Modi, P. R. Ebeling, M. S. Lee, Y. K. Min, A. Mithal, X. Yang, S. Baidya, S. Sen, S. Sajjan

**Affiliations:** 1 Center for Observational and Real-World Evidence (CORE), Merck & Co., Inc, 600 Corporate Drive, Mailstop: CRB-205, Kenilworth, NJ 08833 USA; 2 Monash University, Clayton, Australia; 3 Kaohsiung Chang Gung Memorial Hospital, Chang Gung University, Taoyuan City, Taiwan; 4 Division of Endocrinology and Metabolism, Department of Medicine, Samsung Medical Center, Sungkyunkwan University School of Medicine, Seoul, South Korea; 5 Medanta the Medicity, Gurgaon, Haryana India; 6 Optum, Melbourne, Australia

**Keywords:** Osteoporosis, Postmenopausal, Gastrointestinal diseases, Patient satisfaction, Quality of life, Medication adherence

## Abstract

**Summary:**

The purpose of this study was to describe the impact of gastrointestinal events on patient-reported outcomes and health care resource use among Asia-Pacific women with postmenopausal osteoporosis. The results of this study show that gastrointestinal events decreased adherence, treatment satisfaction, and quality of life in Asia-Pacific women with postmenopausal osteoporosis.

**Purpose:**

This study aimed to describe the impact of gastrointestinal (GI) events on patient-reported outcomes and health care resource use among Asia-Pacific women with postmenopausal osteoporosis.

**Methods:**

The MUSIC OS-AP study included an observational cohort study of postmenopausal women with osteoporosis. Women were classified as untreated or treated, with treated patients further classified as new or experienced users. Adherence was measured by the Adherence Evaluation of Osteoporosis treatment (ADEOS) questionnaire, treatment satisfaction by the Osteoporosis Patient Satisfaction Questionnaire (OPSAT) while general health-related and osteoporosis-specific quality of life were measured by the European Quality of Life-5 Dimensions (EQ-5D) questionnaire and the Osteoporosis Assessment Questionnaire (OPAQ), respectively. The association of GI events with these outcomes was determined by covariate-adjusted regression analysis of least squares mean differences in the scores of treated patients with and without GI events. Resource utilization was measured as the number of physician visits over the past 3 months, and multivariate regression analysis was used to assess the association of GI events with the likelihood of a visit.

**Results:**

The GI event profile, quality of life scores, and resource use were numerically similar in untreated and treated women. The rate of adherence among treated women was higher in experienced than in new users. As indicated by mean scores, experienced users had better quality of life and slightly higher treatment satisfaction and fewer physician visits than new users. Except for adherence in new users, all measures were similarly adversely affected by GI events in both new and experienced users.

**Conclusions:**

GI events decreased adherence, treatment satisfaction, and quality of life in Asia-Pacific women with postmenopausal osteoporosis.

## Introduction

The incidence of osteoporotic hip fracture has risen 2–3-fold in Asia over the past few decades [1]. It is now recognized that the prevalence of osteoporosis in older women (i.e., aged ≥50) living in Asia-Pacific countries is comparable to that in European and North American populations [2]. A scarcity of diagnostic instruments (e.g., dual-energy x-ray absorptiometers) and lack of epidemiologic and economic data have prevented many of these countries from developing official guidelines for diagnosis and treatment of osteoporosis and raising public awareness of the disease [1]. As a result, the burden of osteoporosis in the Asia-Pacific region is not well documented.

In particular, the gastrointestinal (GI) events that are common among users of osteoporosis therapy in US [3] and European [4, 5] populations are virtually uncharacterized in patients in the Asia-Pacific region. Such events, or use of medications suggesting their occurrence, have been shown to be associated with reduced adherence (both compliance and persistence) to treatment [6–11] and low treatment satisfaction [12–14]. Increased health care resource use has also been documented among patients with osteoporosis treatment-related GI events [15, 16]. However, such associations have not been assessed in Asian or Australian populations.

The Medication Use Patterns, Treatment Satisfaction, and Inadequate Control of Osteoporosis Study in the Asia-Pacific Region (MUSIC OS-AP) was designed to address this information gap. The primary objectives of MUSIC OS-AP were (i) to describe the frequency of GI events among postmenopausal women receiving pharmacologic treatment for osteoporosis and (ii) to assess the association between GI events and adherence, treatment satisfaction, health-related quality of life, and health care resource utilization.

## Methods

### Study design

Details of the design of MUSIC OS-AP are given in a previous publication [17]. The study was conducted in five Asia-Pacific countries: Australia, New Zealand, Taiwan, Korea, and India. This manuscript describes patient-reported outcomes from the baseline assessment in the prospective study.

### Study sample

Patients were eligible for enrollment in the prospective study if they were postmenopausal women, at least 50 years of age, had osteoporosis in their physician’s judgment (with or without a BMD test), and provided informed consent. Patients were excluded if they had been diagnosed with Parkinson’s disease or any other neuromuscular disease or Paget’s disease, were currently treated with any injected medication for osteoporosis, had been switched between oral pharmacologic osteoporosis medications within the past 3 months, or were currently or formerly (past 90 days) enrolled in a clinical trial.

### Study definitions

Enrollees were classified as either untreated or treated, with treated patients further classified as new or experienced users. New users were patients who had been receiving oral pharmacologic therapy for less than 3 months, and experience users were patients receiving the same oral pharmacologic therapy for at least 3 months prior to enrollment. Oral pharmacologic therapies included bisphosphonates (e.g., alendronate, risedronate, and ibandronate), calcitonin, strontium ranelate, and selective estrogen-receptor modulators (raloxifene and bazedoxifene).

GI events were defined as the following clinical symptoms: heartburn/acid reflux, upset stomach/indigestion, nausea/vomiting, pain behind the breastbone, pain on swallowing or food sticking, stomach pain above or below the navel, diarrhea or constipation, and bloating.

Patient-reported race was classified as Caucasian, East Asian, or West Asian. Caucasian was defined as European, Mediterranean, Middle Eastern, or North African Descent. East Asian was defined as Chinese, Korean, Japanese, or Taiwanese. West Asian was defined as Indian or Pakistani.

### Study outcomes

GI events were assessed by asking patients whether they had experienced any of the above-listed symptoms in the past 6 months. Answers were indicated with yes/no check boxes for each symptom.

Adherence and treatment satisfaction were assessed in treated patients only. Adherence was measured by the Adherence Evaluation of Osteoporosis treatment (ADEOS) questionnaire [18] and defined as a score of ≥20 out of 22. The questions are not temporally constrained. Patients with missing answers were excluded from the analyses of adherence. Treatment satisfaction was measured with the Osteoporosis Patient Treatment Satisfaction Questionnaire (OPSAT-Q) [19]. The OPSAT-Q consists of 16 items on 4 subscales (convenience, confidence with daily functioning, overall satisfaction, and side effects). Subscale scores are used to create a composite satisfaction score that ranges from 0 to 100, with higher scores indicating greater treatment satisfaction. The questions have no temporal frame of reference.

Quality of life and health care resource use were assessed in both untreated and treated patients. General health-related quality of life was measured with the EuroQol-5D-3L [20]. The EuroQol-5D has two components: a utility score (scale 0 to 1.0, where 1.0 is defined as full health, zero as death) and a visual analog scale (VAS; scale 0–100, 100 being best imaginable health). The utility score comprises five dimensions (mobility, self-care, usual activities, pain/discomfort, and anxiety/depression) and is not bounded by time, and the VAS asks about the patient’s overall quality of life “today.” The minimal clinically important difference in the utility score, defined as the smallest change which patients perceive as beneficial and which would mandate a change in disease management, has been reported alternatively as 0.074 [21] and 0.03 [22]. Osteoporosis-specific quality of life was measured with the short version of the Osteoporosis Assessment Questionnaire (OPAQ-SV) [23]. The OPAQ-SV consists of 34 items in three domains (physical function, emotional status, and back pain), each with a scale of 0–100. Higher scores indicate better quality of life. The time frame of the questionnaire is the previous 2 weeks. Analyses of the physical function domain have suggested that a difference of 10 points would be clinically meaningful at the individual patient level [24]. Health care resource utilization was measured as the number of osteoporosis-related primary care and specialist physician visits over the past 3 months.

All questionnaires were made available in the local language of the participating clinics (see the original MUSIC OS-AP publication for more details [17]).

### Statistical analysis

Demographic and clinical characteristics and patient-reported outcomes were analyzed descriptively. Continuous variables were reported as means and standard deviations and categorical variables as numbers and percentages.

The effect of GI events on patient-reported outcomes at baseline was assessed in treated patients by calculating the differences in covariate-adjusted least-squares mean scores between patients with and without GI events. Covariates used to adjust the scores were age, age at menopause, highest level of education (university degree or higher, non-university certificate or diploma, high school, did not finish high school, or prefer not to answer), country of residence (Australia/New Zealand, Korea, Taiwan, or India), body mass index, history of GI events (yes or no), user status (new or experienced), number of previous fractures, number of previous falls, predominant treatment in the 12 months before baseline (bisphosphonate, non-bisphosphonate, bisphosphonate/non-bisphosphonate combination, or no medication), hours of physical exercise per week, presence of at least one risk factor (yes or no; risk factors were parental hip fracture, current smoking, glucocorticoid use, and alcohol consumption ≥3 units per day), presence of comorbidities (yes or no; comorbidities were rheumatoid arthritis, secondary osteoporosis, diabetes, chronic kidney disease, etc.), and a term for the interaction between history of GI events and user status. These same variables were used to adjust a multivariate regression analysis in which the association of GI events with the likelihood of each type of health care resource use was assessed. In all analyses, a *P* value of <0.05 was considered statistically significant.

## Results

### Characteristics of untreated patients

A total of 300 untreated patients were enrolled in the study (Table 1). Their mean age was 63.0 years, and the majority (56.7%) were West Asian. Approximately one fifth had a history of osteoporotic fracture (17.7%) or a fall within the previous 12 months (21.7%). Most untreated patients (61.3%) reported experiencing a GI event in the past 6 months.

**Table 1:** Demographic and clinical characteristics of the study population

	Untreated (*N* = 300)	All treated patients (*N* = 3286)	New users (*N* = 1416)	Experienced users (*N* = 1870)
Age, mean (SD) years	63.0 (9.3)	65.4 (9.5)	62.9 (9.5)	67.3 (9.0)
Age at menopause, mean (SD) years	48.5 (4.8)	47.9 (5.4)	47.2 (5.6)	48.4 (5.1)
Race^a^				
Caucasian	36 (12.0)	454 (13.8)	80 (5.6)	374 (20.0)
East Asian	97 (32.3)	1479 (45.0)	372 (26.3)	1107 (59.2)
West Asian	167 (56.7)	1352 (41.1)	963 (68.0)	389 (20.8)
Other	0 (0.0)	1 (0.03)	1 (0.07)	0 (0.0)
History of OP fracture	53 (17.7)	707 (21.5)	225 (15.9)	482 (25.8)
Falls in the past 12 months	65 (21.7)	593 (18.0)	262 (18.5)	331 (17.7)
GI events in the past 6 months	184 (61.3)	1967 (59.9)	814 (57.5)	1153 (61.7)
Upper GI	154 (51.3)	1658 (50.5)	720 (50.8)	938 (50.2)
Heartburn/acid reflux	103 (34.3)	1098 (33.4)	455 (32.1)	643 (34.4)
Upset stomach/indigestion	75 (25.0)	816 (24.8)	363 (25.6)	453 (24.2)
Nausea/vomiting	44 (14.7)	377 (11.5)	152 (10.7)	225 (12.0)
Pain behind the breastbone	23 (7.7)	375 (11.4)	172 (12.1)	203 (10.9)
Pain on swallowing or food sticking	17 (5.7)	227 (6.9)	102 (7.2)	125 (6.7)
Stomach pain above the navel	28 (9.3)	260 (7.9)	107 (7.6)	153 (8.2)
Lower GI	96 (32.0)	863 (26.3)	347 (24.5)	516 (27.6)
Diarrhea or constipation	86 (28.7)	779 (23.7)	305 (21.5)	474 (25.3)
Stomach pain below the navel	26 (8.7)	227 (6.9)	93 (6.6)	134 (7.2)
Bloating	68 (22.7)	687 (20.9)	264 (18.6)	423 (22.6)
Osteoporosis medication				
Bisphosphonates	–			
Alendronic acid	–	1695 (51.6)	371 (26.2)	867 (46.4)
Etidronic acid	–	2 (0.06)	0 (0.0)	2 (0.11)
Ibandronic acid	–	339 (10.3)	73 (5.2)	162 (8.7)
Pamidronic acid	–	1 (0.03)	0 (0.0)	1 (0.05)
Risedronic acid	–	872 (26.5)	129 (9.1)	533 (28.5)
Non-bisphosphonates				
Bazedoxifene	–	66 (2.0)	19 (1.3)	47 (2.5)
Calcitonin	–	7 (0.21)	2 (0.14)	5 (0.27)
Denosumab	–	1 (0.03)	0 (0.0)	1 (0.05)
Raloxifene	–	299 (9.1)	44 (3.1)	255 (13.6)
Strontium ranelate	–	55 (1.7)	7 (0.49)	48 (2.6)
Teriparatide	–	4 (0.12)	1 (0.07)	3 (0.16)
Adherence^b^	–	943 (38.1)	202 (33.6)	741 (39.6)
OPSAT-Q score, mean (SD)^c^	–	79.1 (12.9)	78.1 (13.7)	79.5 (12.6)
EQ-5D-3L utility score, mean (SD)^d^	0.70 (0.28)	0.71 (0.28)	0.62(0.30)	0.77 (0.24)
EQ-5D-3L VAS score, mean (SD)^d^	66.8 (18.7)	67.6 (18.5)	62.8 (17.6)	71.2 (18.3)
OPAQ-SV scores, mean (SD)^d^				
Physical function	66.7 (18.6)	67.6 (19.9)	59.5 (20.0)	73.8 (17.4)
Emotional status	65.1 (21.6)	65.4 (19.5)	62.4 (19.8)	67.7 (18.9)
Back pain	59.1 (28.9)	65.7 (26.7)	58.8 (27.3)	70.9 (25.0)
Primary care visits, mean (SD)^e^	1.5 (0.95)	1.5 (0.93)	1.7 (1.10)	1.4 (0.75)
Specialist visits, mean (SD)^e^	1.6 (0.82)	1.5 (0.92)	1.7 (1.01)	1.4 (0.84)

*EQ-5D-3L* European Quality of Life-5 Dimensions, *GI* gastrointestinal, *OP* osteoporosis, *OPAQ-SV* Osteoporosis Assessment Questionnaire, *OPSAT-Q* Osteoporosis Patient Satisfaction Questionnaire, *SD* standard deviation, *VAS* visual analog scale

Values are presented as *N* (%) unless otherwise indicated

^a^Caucasian was defined as European, Mediterranean, Middle Eastern, or North African Descent. East Asian was defined as Chinese, Korean, Japanese, or Taiwanese. West Asian was defined as Indian or Pakistani. Other included only those identifying as New Zealand Maori

^b^Calculated for patients responding to this survey question. Total *N* = 2471 (experienced users = 1869; new users = 602)

^c^Calculated for patients responding to this survey question. Total *N* = 2529 (experienced users = 1867; new users = 662)

^d^Averaged over all available data. Missing data constituted <0.5% of patients

^e^Averaged among patients with any primary care visits (*N* = 51, 634, 291, and 343, respectively) or specialist visits (*N* = 96, 1255, 506, and 749, respectively)

Mean scores on the EQ-5D-3L were 0.70 for the utility measure and 66.8 for the VAS (Table 1). Mean scores on the OPAQ-SV were 66.7 for physical function, 65.1 for emotional status, and 59.1 for back pain. Untreated patients reported an average of 1.5 osteoporosis-related primary care visits and 1.6 osteoporosis-related specialist visits in the previous 3 months.

### Characteristics of treated patients

A total of 3286 treated patients were enrolled in the study (Table 1). The mean age of treated patients was 65.4 years, and there was a nearly equal representation of East Asians (45.0%) and West Asians (41.1%). Approximately one fifth had a history of osteoporotic fracture (21.5%) or had had a fall within the past 12 months (18.0%). Most treated patients (59.9%) reported having GI events in the previous 6 months. In the past 12 months, 88.1% of all treated patients reported using a bisphosphonate while 13.1% reported using a non-bisphosphonate (Table 1).

Only 38.1% of the treated patients qualified as adherent with their treatment, and the mean satisfaction score was 79.1 (Table 1). Mean scores on the EQ-5D-3L were 0.71 for the utility measure and 67.6 for the VAS. Mean scores on the OPAQ-SV were 67.6 for physical function, 65.4 for emotional status, and 65.7 for back pain. Treated patients reported an average of 1.5 osteoporosis-related primary care visits and 1.5 osteoporosis-related specialist visits in the previous 3 months.

Among the treated patients, 1416 were new users and 1870 were experienced users of osteoporosis treatment (Table 1). New users were younger (mean age 62.9) than experienced users (mean age 67.3), and the majority of them were West Asian (68.0%), whereas the majority of experienced users were East Asian (59.2%). A history of osteoporotic fracture was more prevalent among experienced users (25.8%) than new users (15.9%). More experienced users (39.6%) were adherent with treatment than new users were (33.6%), but their satisfaction levels were approximately equal. All quality of life scores were better in experienced users than in new users, and osteoporosis-related resource use was less frequent in experienced users than in new users (Table 1).

### Effect of GI events on patient-reported outcomes

Differences in least-squares mean scores between treated patients with and without GI events are shown in Table 2. Least squares mean differences associated with GI events in all treated patients were −0.348 for the adherence score, −6.940 for the OPSAT score, −0.092 for the EQ-5D utility score, −4.614 for the EQ-5D visual analog scale score, −3.663 for the OPAQ physical function score, −4.435 for the OPAQ emotional status score, and −8.183 for the OPAQ back pain score. All these differences met the criterion for statistical significance. Expressed as a percentage of the total scale for each outcome, the differences associated with GI events ranged from 1.6% for adherence to 9.2% for the EQ-5D utility score. Except for adherence in new users, all of these measures were significantly adversely affected by GI events in both new and experienced users (Table 2). The decrement in treatment satisfaction associated with GI events was significantly greater in new users than in experienced users (CI for new users −10.45 to −6.834; CI for experienced users −6.350 to −4.126; Table 2). For all other outcomes, new and experienced users were affected to a similar extent.

**Table 2:** Least-squares mean differences between treated patients with and without GI events at baseline

	With GI events: LS mean	Without GI events: LS mean	LS mean difference	95% CI	*P* value
Adherence					
All treated	18.155	18.503	−0.348	−0.591, −0.104	0.005
New users	17.634	17.825	−0.191	−0.609, 0.227	0.370
Experienced users	18.676	19.181	−0.504	−0.750, −0.258	<0.001
OPSAT-Q score					
All treated	76.323	83.263	−6.940	−8.007, −5.874	<0.001
New users	74.020	82.662	−8.643	−10.45, −6.834	<0.001
Experienced users	76.437	83.864	−5.238	−6.350, −4.126	<0.001
EQ-5D-3L utility score					
All treated	0.707	0.799	−0.092	−0.109, −0.074	<0.001
New users	0.674	0.777	−0.103	−0.129, −0.077	<0.001
Experience users	0.740	0.821	−0.080	−0.103, −0.057	<0.001
EQ-5D-3L VAS score					
All treated	68.120	72.734	−4.614	−5.795, −3.433	<0.001
New users	67.862	72.341	−4.479	−6.235, −2.723	<0.001
Experienced users	68.378	72.345	−4.748	−6.307, −3.189	<0.001
OPAQ-SV physical function score					
All treated	67.874	71.536	−3.663	−4.763, −2.563	<0.001
New users	65.798	69.783	−3.985	−5.621, −2.349	<0.001
Experienced users	69.950	73.290	−3.340	−4.793, −1.888	<0.001
OPAQ-SV emotional status score					
All treated	63.707	68.141	−4.435	−5.690, −3.180	<0.001
New users	62.759	66.132	−3.373	−5.240, −1.507	<0.001
Experienced users	64.654	70.150	−5.496	−7.153, −3.839	<0.001
OPAQ-SV back pain score					
All treated	66.572	74.754	−8.183	−9.888, −6.478	<0.001
New users	63.692	72.616	−8.924	−11.46, −6.388	<0.001
Experienced users	69.451	76.893	−7.442	−9.693, −5.191	<0.001

Scores were adjusted by multivariate regression for the following variables: age, age at menopause, highest level of education, country, BMI, history of GI events, user status (among all treated patients), number of previous fractures, number of previous falls, predominant treatment, hours of physical exercise per week, presence of at least one risk factor, presence of comorbidities, and interactions between history of GI events and treatment group

*CI* confidence interval, *EQ-5D-3L* European Quality of Life-5 Dimensions, *GI* gastrointestinal, *LS* least squares, *OPAQ-SV* Osteoporosis Assessment Questionnaire, *OPSAT-Q* Osteoporosis Patient Satisfaction Questionnaire, *VAS* visual analog scale

In regression analyses of resource use (Table 3), experienced users with GI events were 43% more likely to have a primary care visit than were those without GI events (OR 1.43, 95% CI 1.08–1.9). GI events were not associated with primary care visits in new users or all treated patients and had no association with specialist visits.

**Table 3:** Likelihood of osteoporosis-related health care resource use among treated patients with versus without GI events at baseline

	Odds ratio	95% CI	*P* value
Primary care visits			
All treated	1.11	0.91–1.35	0.32
New users	0.86	0.65–1.13	0.27
Experienced users	1.43	1.08–1.89	0.01
Specialist visits			
All treated	0.95	0.82–1.11	0.54
New users	0.99	0.79–1.25	0.95
Experience users	0.92	0.75–1.12	0.38

Odds ratios were adjusted for the following variables: age, age at menopause, highest level of education, country, BMI, history of GI events, user status (among all treated patients), number of previous fractures, number of previous falls, predominant treatment, hours of physical exercise per week, presence of at least one risk factor, presence of comorbidities, and interactions between history of GI events and treatment group

*CI* confidence interval

## Discussion

This baseline assessment of the MUSIC OS-AP population showed that GI events decreased adherence to treatment, treatment satisfaction, and quality of life, as reported by Asia-Pacific women receiving pharmacologic treatment for postmenopausal osteoporosis. GI events did not affect osteoporosis-related resource use.

There is little information on the effect of GI events on adherence to osteoporosis therapy in the Asia-Pacific region. In a study of 208 Japanese osteoporosis patients (1.5% male), patient-reported side effects were the primary driver of non-compliance with alendronate or risedronate [25]. Another study of 1307 Japanese patients (15.1% male) receiving their first bisphosphonate at a university hospital found that switching therapies because of adverse effects was associated with reduced persistence and that discontinuation of the first drug due to adverse effects was associated with higher rates of discontinuation of the second drug for the same reason (hazard ratio 4.2, 95% CI 2.1–8.4) [26]. However, neither of these studies limited the analysis of adverse events to GI events. Previous US studies have reported the effects of GI events on adherence among osteoporosis patients; however, these studies did not assess the effect of GI events on adherence using the ADEOS questionnaire [6, 10, 11, 13]. Among these US studies, the Prospective Observational Scientific Study Investigating Bone Loss Experience (POSSIBLE) study is best suited for comparison to MUSIC OS [13]. In the US cohort of POSSIBLE, women reporting GI side effects were more likely to discontinue therapy at both 6 and 12 months [13]. However, discontinuation is not directly comparable to the medication-taking behaviors assessed by the ADEOS questionnaire. Such behaviors were assessed using ADEOS by the MUSIC OS study in Europe and Canada (MUSIC OS-EU), which found that treated osteoporosis patients with GI events had lower ADEOS scores than those without GI events (adjusted least squares mean difference −0.43; *P* < 0.001) [27]. This result is consistent with the findings of the MUSIC OS-AP study (adjusted least squares mean difference −0.348). The MUSIC OS-AP study is unique in that it directly assesses the association of GI events with adherence to oral osteoporosis therapies in the Asia-Pacific region using ADEOS scores.

The relationship of GI events to treatment satisfaction was assessed with the OPSAT questionnaire in a study of 4220 Korean women taking oral bisphosphonates [28]. The mean composite OPSAT score was 75.3 in non-users of acid-related medications versus 70.8 in users of such medications. Taking acid-related medication use as a proxy for GI symptoms, these data are quite similar to ours. In MUSIC OS-AP, the mean composite OPSAT score at baseline was 79.1 among treated patients, and GI events were shown to reduce this score by 6.9 points. We identified only one other study from Asia reporting the effects of GI symptoms on treatment satisfaction. Chung et al. used a crossover design to assess patient preferences for monthly ibandronate versus weekly risedronate in 365 Korean women [29]. In this study, less stomach discomfort and more tolerable side effects were among the patient-reported reasons for a preference for the ibandronate regimen. In the MUSIC OS-EU study, covariate-adjusted OPSAT scores of experienced users of treatment for osteoporosis were 5.68 points lower in those with versus without GI events [27], a decrement comparable to that seen in experienced users in the current study (−5.24). In the POSSIBLE-US cohort, women experiencing a GI side effect at month 6 had lower global treatment satisfaction scores (assessed by the Treatment Satisfaction Questionnaire for Medication) than those without GI side effects [13].

In MUSIC OS-AP, both general and osteoporosis-specific quality of life were reduced by GI events. Least-squares mean differences associated with GI events were −0.092 for the EQ-5D utility score and −3.663 for the OPAQ physical function score. Although both values were statistically significant, only the former met the threshold of the minimal clinically important difference (see the “Methods” section). Previous studies of the relationship of GI events and quality of life are few. In one of the Korean studies described above, osteoporosis-specific quality of life was reduced in patients using acid-related medications (a proxy for GI events); the quality of life domain scores on the OPSAT questionnaire were 62.7 in users and 66.8 in non-users of acid-related medications (*P* < 0.001) [28]. Similar to the results reported in MUSIC OS-AP, EQ-5D and OPAQ-SV physical function scores were significantly reduced in patients with versus without GI events in the MUSIC OS-EU study [27]. In contrast, in POSSIBLE-US, EQ-5D utility scores measured at month 6 were not significantly different between patients with and without GI side effects [13].

The MUSIC OS-AP study included untreated patients at the baseline assessment, 18% of whom had a history of osteoporotic fracture. This fracture rate is somewhat lower than the expected lifetime fracture risk of 30 to 40% for older women [30–32]. The GI event rate in this population (61%) is consistent with previous reports that GI symptoms are common in Asian adults, regardless of osteoporosis treatment [33–36]. The EQ-5D-3L utility and VAS scores in untreated osteoporosis patients in MUSIC OS-AP (0.70 and 66.8, respectively) were lower than those in the published population norms for women in Australia (0.87 utility) [37], New Zealand (0.85 and 81.2, respectively) [38], Taiwan (74.3 VAS) [39], and Korea (0.89 and 78.7, respectively) [38]. GI event rates, quality of life scores, and resource use were numerically similar in untreated and treated women.

The results of the current MUSIC OS-AP analysis improve upon previous studies in several ways. First, MUSIC OS-AP assessed quality of life and treatment satisfaction separately in patients with or without GI events, a design element missing from earlier studies of this question [12, 14]. Second, our analyses were adjusted for demographic and clinical covariates, such that the results indicate an effect of GI events on adherence, quality of life, and treatment satisfaction independent of confounder variables. Finally, to our knowledge, MUSIC OS-AP is the first study to assess directly the association of GI events with adherence, quality of life, and treatment satisfaction in osteoporosis patients from the Asia-Pacific region.

Despite these strengths, the current analysis is subject to several important limitations. First, due to the design of the study as a patient survey, the accuracy of the findings is limited by patient recall and potentially affected by reporting bias. Second, the least-squares mean differences were not adjusted for adherence, so some patients may have had GI events not associated with treatment. Third, patients were not required to report the severity of the GI events; in fact, GI events typically considered severe (e.g., those involving bleeding or perforation) were not included in the patient questionnaire. Thus, our results reflect primarily the experience of patients with mild to moderate GI symptoms. Fourth, the lack of statistical comparisons of untreated and treated patients precludes drawing conclusions regarding the relationship between treatment and GI event rates and quality of life. Fifth, the data were pooled from culturally and demographically different countries and therefore reflect the average effect within potentially disparate data. Finally, lack of information about the minimal clinically important difference on the ADEOS and OPSAT questionnaires prevents assessment of the clinical relevance of the effect sizes reported here, and some of the observed differences in quality of life, although statistically significant, may not have been clinically important.

In conclusion, the baseline assessment of MUSIC OS-AP showed that GI events were common in postmenopausal women with osteoporosis in the Asia-Pacific region, occurring in approximately 60% of both treated and untreated patients. Among women treated for osteoporosis, GI events were associated with decreased adherence, lower treatment satisfaction, and worse quality of life. Treatment satisfaction was affected to a greater extent in new users of osteoporosis treatment versus experienced users. These results suggest that GI events should be given consideration during the clinical management of osteoporotic women in the Asia-Pacific region.
